# Resistance and tolerance to the brown planthopper, *Nilaparvata lugens* (Stål), in rice infested at different growth stages across a gradient of nitrogen applications

**DOI:** 10.1016/j.fcr.2017.12.008

**Published:** 2018-03

**Authors:** Finbarr G. Horgan, Ainara Peñalver Cruz, Carmencita C. Bernal, Angelee Fame Ramal, Maria Liberty P. Almazan, Andrew Wilby

**Affiliations:** a University of Technology Sydney, 15 Broadway, Ultimo, Sydney, NSW 2007, Australia; b International Rice Research Institute, DAPO Box 7777, Metro Manila, Philippines; c Lancaster Environment Centre, Lancaster University, Lancaster, LA1 4YQ, UK

**Keywords:** Anti-feeding, Herbivory, Host plant resistance, Ontogenetics, Phloem-feeding, Plant vigour hypothesis

## Abstract

High resource availability can reduce anti-herbivore resistance (a plant’s ability to defend against herbivores and reduce damage) in rice, *Oryza sativa* L, but may also increase tolerance (a plant’s ability to withstand damage by, for example, compensatory growth). Through a series of greenhouse, screenhouse and field experiments, this study examines fitness (survival and development × reproduction) of the brown planthopper, *Nilaparvata lugens* (Stål), on resistant (IR62) and susceptible (IR22) rice varieties and age-related rice tolerance to planthopper damage under varying resource (nitrogenous fertilizer) availability. Planthoppers reared on IR62 in the greenhouse had lower fitness than planthoppers on IR22. IR62 became increasingly resistant as plants aged. IR22 was generally more tolerant of planthopper damage, and tolerance increased in IR22, but declined in IR62, as the plants aged. Rice plants infested at pre-tillering stages (3–4 leaf stage) in the screenhouse had greater losses to root, shoot and grain yield per unit weight of planthopper than plants infested at tillering stages, particularly in IR22. These trends were mainly due to the impact of planthoppers during pre-tillering stages and the length of exposure to the planthoppers. High nitrogen compromised IR62 resistance, particularly in tillering plants in the greenhouse study; however, high nitrogen did not increase planthopper biomass-density on IR62 in greenhouse or field cages. Tolerance to damage in IR62 at mid-tillering stages declined under increasing levels of nitrogen, but nitrogen increased tolerance during late-tillering stages. Planthopper damage to IR22 in field cages was severe and hopperburn (plant death) occurred in 83% of IR22 plants under high nitrogen (60–150 kg N ha^−1^). In contrast, despite planthopper infestations, damage to IR62 was low in field-grown plants and productivity (tillers, roots, shoots and grain) increased in IR62 under increasing nitrogen. Our results indicate that, whereas nitrogenous fertilizer increases planthopper fitness on susceptible and resistant varieties, the net effects of high nitrogen on IR62 include decreased planthopper biomass-density (apparent in all experiments) and higher tolerance to damage during later growth stages (observed in the greenhouse, and during one of two seasons in field cages).

## Highlights

• IR62 planthopper resistance increased but tolerance decreased from pre- to late-tillering stages.
• Planthopper tolerance in the susceptible IR22 increased from pre-tillering to tillering stages.
• High nitrogen decreased resistance of IR62 in the greenhouse but not in field plots.
• High nitrogen increased tolerance in IR62, but IR22 was more heavily damaged.

## 1 Introduction

Vigorously growing plants, such as plants growing under nutrient-rich and high-light conditions, are often more attractive to herbivores (plant vigour hypothesis, PVH: Price, 1991). In a meta-analysis by Cornelissen and Fernandes (2008) of 71 published studies, phloem-feeding herbivores (Hemiptera) in particular, were shown to increase in abundance (up to 75%) on more vigorous plants or modules, in effect, decreasing anti-herbivore resistance (the ability to defend against herbivores: Table 1). Since tolerance to herbivory (a plant’s ability to withstand herbivore attack by, for example compensatory growth: Table 1) is often higher under high resource/low competition conditions (compensatory continuum hypothesis, CCH: Maschinski and Whitham, 1989), vigorous plants are also expected to better tolerate herbivore damage than less vigorous plants. This has been demonstrated across a range of wild and cultivated plants (Müller-Schärer et al., 2004, Wise and Abrahamson, 2007, Lv et al., 2008, Horgan et al., 2017). As evidence accumulates, a number of exceptions to these general trends have become apparent (Huberty and Denno, 2006, Rubia-Sanchez et al., 2003, Butler et al., 2012, Horgan et al., 2016a), putting into question the predictive value of general hypotheses about plant-herbivore interactions (Wise and Abrahamson, 2005, Wise and Abrahamson, 2007). Identifying the mechanisms that underlie such exceptions will improve knowledge of the dynamic nature of herbivore-plant interactions and help define practical applications for herbivore management in agricultural systems.

**Table 1:** Functional categories of plant-herbivore interaction.^a^

Functional category	Definition	Effects on herbivores^b^	Effects on plant^b^	Estimated in this study
Susceptibility	Inability of a plant to deter or defend against damage from herbivores	No negative effects	Normally high damage	Comparison of damage (biomass loss) against resistant variety
Resistance	A plant trait that reduces potential damage from herbivores compared to susceptible plants	Relative decline in fitness through antixenotic or antibiotic effects^c^	Relative decline in damage	Comparison of damage (biomass loss) against susceptible variety
Antixenosis	Plant traits (mechanisms) that deter herbivores from ovipositing or from initiating feeding	Relatively low oviposition and/or feeding preferences	Relative decline in damage	Not estimated here (requires choice bioassays)
Antibiosis	Plant traits (mechanisms) that adversely affect the survival, growth or reproductive output of herbivores	Relative decline in survival, biomass, development time, or fecundity	Relative decline in damage	Comparisons of oviposition, nymph survival, nymph biomass, development times, and feeding efficiency (honeydew) with susceptible variety
Tolerance	A plant's capacity to withstand herbivore damage and continue to grow and/or yield satisfactorily during and after herbivore attack	No negative effects	Maintains relatively high growth rates (biomass) or reproductive output (yield) per unit of herbivore biomass^d^	Estimated as the relative decline in plant fitness per unit weight of planthopper across a gradient of environments

a For further details see Strauss and Agrawal (1999) and Smith (2005).

b Comparative effects relative to a susceptible variety.

c Fitness is a quantitative representation of reproductive success (genetic contribution to future generations through survival and development × reproductive output) in a given environment.

d Observed during low levels of intraspecific competition between herbivores.

Rice, *Oryza sativa* L, is a useful model for understanding resource effects on herbivore-plant interactions. This is because, as a grass, rice is a typical modular plant that continuously produces tillers during the vegetative growth phase (Counce et al., 2000). Rice is also a well-researched agricultural crop with a diverse assemblage of associated insect herbivores (Heinrichs, 1994a, Heinrichs, 1994b, Heinrichs and Barrion, 2004). When grown under high nitrogen conditions, rice is more attractive to a range of herbivores and diseases (Visarto et al., 2001, Jiang and Cheng, 2003, Hu et al., 2016). Rice also compensates well for herbivore damage, often with increased tolerance under higher nitrogen conditions (Rubia-Sanchez et al., 1999, Reay-Jones et al., 2008, Horgan et al., 2016a). However, in a recent study, Horgan et al. (2016a) identified some exceptions to these trends. In particular, although nitrogen increased rice tolerance to the whitebacked planthopper, *Sogatella furcifera* (Horváth), and yellow stem borer, *Scirpophaga incertulas* (Walker), in their experiments, these authors noted the opposite effect in plants infested with the brown planthopper, *Nilaparvata lugens* (Stål). It appeared that the brown planthopper, which is often regarded as the most serious pest of rice in Asia, is able to pre-empt extra available nutrients from the plant phloem before these can be used by the host plant – thereby decreasing plant tolerance under high nitrogen (Horgan et al., 2016a).

For the last several decades, research into host plant resistance has dominated literature on the management of rice planthoppers (Fujita et al., 2013). This research has greatly increased knowledge of planthopper-rice interactions and has identified over 30 potentially useful resistance gene loci as well as several major quantitative trait loci (Fujita et al., 2013). However, it is clear that target planthoppers can rapidly adapt to resistant varieties and genes and that several resistance sources (either donor varieties or genes) are now no longer effective over much of the planthopper range (Tanaka and Matsumura, 2000, Myint et al., 2009, Horgan et al., 2015). Studies have also indicated that host plant resistance is influenced by crop management practices, particularly the use of pesticides (Gallagher et al., 1994) and fertilizers (Salim and Saxena, 1991, Jiang and Cheng, 2003, Horgan et al., 2016b). Few studies have incorporated crop management as a contributing factor in the successful resistance of rice against planthoppers and other insect herbivores in the field (but see Cuong et al., 1997, Visarto et al., 2001, Zhu et al., 2004).

Successful deployment of resistant rice varieties is further complicated by a gap in the current understanding of how planthopper-rice interactions change over the course of plant development (often referred to as ontogenetics). Since plants face distinct challenges as they grow and develop, they are predicted to shift their defence strategies to counter their most probable biotic stresses (Price, 1991, Herms and Mattson, 1992). Resistance to planthoppers in rice may increase or decrease during plant development (Rapusas and Heinrichs, 1982, Baqui and Kershaw, 1993, Vu et al., 2014, Horgan et al., 2016b) presumably depending on underlying resistance mechanisms or resistance genes (Srinivasan et al., 2015, Srinivasan et al., 2016). Therefore, the age at which plants are attacked, how resistance changes over plant development and how farmers manage their crop (vis-à-vis fertilizer and pesticide inputs) represent key determinants of planthopper population growth and damage responses in fields of resistant rice.

In the present study, we examine the integrity of rice resistance under a range of nitrogen levels through a series of greenhouse, screenhouse and field experiments. We also examine how nitrogen affects ontogenetic changes in resistance and tolerance to planthoppers during crop development. In our experiments, we compared herbivory on the resistant rice variety IR62 against herbivory on the susceptible variety IR22. We used IR62 in our experiments as a modern variety with noted and consistently high resistance to planthoppers from recent screening in South and South East Asia (Horgan et al., 2015), and because the variety possesses resistance genes derived from PTB33, which is a popular source of resistance in modern rice breeding programs (Liu et al., 2015, Ren et al., 2016). Based on optimal defence theory (Herms and Mattson, 1992, Van Dam et al., 1996) and the assumption that trade-offs exist between tolerance and resistance (Núñez-Farfán et al., 2007), we hypothesized that rice plants will be least resistant to planthoppers during actively tillering vegetative stages and gain resistance but lose tolerance as plants age (e.g., Rubia et al., 1989, Price, 1991, Rubia-Sanchez et al., 1999). Furthermore, we predicted that resistance would decrease under high nitrogen as the nutritive value of the plants increases and planthoppers gain more resources from their feeding efforts (Sogawa, 1982, Hu et al., 2016), but that tolerance to planthoppers would increase under high nitrogen because of faster growth rates (CCH) and negative relationships between tolerance and defence. We expected that a resource-related increase in tolerance would be most apparent in IR62 because of inefficient planthopper feeding on this resistant variety (Horgan et al., 2016c). We discuss our results in the light of sustainably deploying resistant rice varieties as part of integrated approaches to planthopper management.

## 2 Materials and methods

### 2.1 Insect herbivores

We focused our experiments on the brown planthopper, which is one of the most damaging rice pests in Asia (Bottrell and Schoenly, 2012). In our experiments, we used planthoppers from a colony maintained at the International Rice Research Institute (IRRI) in the Philippines. The colony was initiated in 2009 with >500 wild caught individuals from Laguna Province (Philippines: 14°10′N, 121°13′E). Planthoppers from the region have noted virulence against a range of resistance genes including *Bph1*, *bph2*, *bph5*, *bph7*, *bph8*, *Bph18*, *BPH25* and *BPH26* (Horgan et al., 2015). The insects were reared continuously on the susceptible variety TN1 (≥30-day old rice plants) in wire mesh cages (91.5 × 56.5 × 56.5 cm; H × L × W). The colony was kept under greenhouse conditions (26–37 °C, 12:12 day:night [D:N]) with feeding plants replaced every 3–5 days. During our field experiments, we also collected data on other planthopper and leafhopper species. Among these, the whitebacked planthopper and green leafhopper, *Nephotettix virescens* (Distant), were most abundant.

### 2.2 Plant materials

We used the resistant rice variety IR62 in our experiments. IR62 is one of few modern rice varieties with demonstrated and consistent resistance to planthoppers from recent greenhouse and field studies (Azzam and Chancellor, 2002, Peñalver-Cruz et al., 2011, Horgan et al., 2015). There is strong evidence that IR62 acquired resistance from the Indian donor variety PTB33 (Khush and Virk, 2005, Liu et al., 2015, Ren et al., 2016). PTB33 possesses at least three genes for resistance to the brown planthopper (*Bph32*, *Bph3*(t) and *BPH26* [a synonym with *bph2*]: Ren et al., 2016). The *Bph32* and *BPH26* genes have recently been cloned (Tamura et al., 2014, Ren et al., 2016). It is still unclear which resistance genes have been introgressed from PTB33 to IR62, although previous studies indicate that resistance in the variety is due to either the *Bph3* locus or *Bph32* gene (and not *BPH26*) (e.g., Peñalver-Cruz et al., 2011, Horgan et al., 2015). Although the underlying genetic mechanisms of rice resistance are often well researched, knowledge of the actual biochemical, nutritive, anatomical or physiological resistance mechanisms associated with most resistant varieties and resistance genes is frequently lacking (Fujita et al., 2013). In contrast, clear planthopper resistance mechanisms have been identified among varieties that had PTB33 or the closely related variety Rathu Heenati (which also possesses the *Bph3* locus) as resistance donors (Stevenson et al., 1996, Liu et al., 2015). Furthermore, DNA sequencing and detailed expression analysis suggest that *Bph32* plays a role in inhibiting feeding by the planthopper (Ren et al., 2016). Although not widely planted, IR62 has been popular in Mindanao (southern Philippines) and Cambodia, largely due to its apparent resistance to the green leafhopper and its capacity to reduce the incidence of tungro virus (Azzam and Chancellor, 2002, Peñalver-Cruz et al., 2011). We used the variety IR22 in our experiments to represent a modern susceptible variety for comparison. IR22 has no known resistance to planthoppers. The variety has been reported to express similar phenological and morphological development to IR62 (i.e., 118–120 days to mature, 98 cm high, 14 tillers per hill: Khush and Virk, 2005, see also Table S1). Seed of the two varieties was acquired through the IRRI Germplasm Collection. The development rates of IR22 and IR62 were largely similar to each other during all experiments described here (IR62 had higher tillering, Table S1) but differed among experiments.

### 2.3 Experimental design

We conducted a series of experiments to examine how the phenological stages at which plants are infested affect resistance and tolerance to the brown planthopper under gradients of added nitrogenous fertilizer. These experiments were conducted under greenhouse, screen house and field conditions. Greenhouse experiments are useful to compare planthopper fitness responses (survival and development × reproduction) to resistant and susceptible plants under low herbivore pressure. In greenhouse bioassays, severe damage to the plants, which would confound response estimates, is avoided because only small numbers of nymphs or adults are normally used. Using plants grown at staggered intervals, such short-duration bioassays can be used to estimate ontogenetic changes in resistance and tolerance. High herbivore densities are often required to examine responses by plants to sustained herbivory (over multiple plant stages and herbivore generations). In such long duration bioassays, plants can become rapidly pot-bound (Crisol et al., 2013); therefore, we also grew rice plants in large pots in a screenhouse to compare plant responses to attacks by planthoppers at different growth stages. Finally, we conducted a field study during two seasons to assess the agronomic performance of the varieties, to verify the resistance of IR62 under more representative rice production conditions, and to examine tolerance in plants with relatively unrestricted root development (i.e., not grown in pots).

#### 2.3.1 Greenhouse experiment

Ontogenetic changes in planthopper nymph and egg-laying responses to IR62 and IR22 under three nitrogen levels were examined in the greenhouse. Temperatures ranged between 26 °C and 37 °C and no artificial lighting was used (12:12 h, D:N). For each bioassay, 54 size-6 pots (15 × 15 cm: height × diameter [H × D]) were filled with paddy soil. These were divided into three groups of 18 pots. Two groups of pots were treated with basal applications of ammonium sulphate to simulate field applications equivalent to 60 or 150 kg N ha^−1^. The amounts of fertilizer added to the pots were calculated based on the estimated weight of top soil in a hectare of paddy (2000 t), the weight of soil in the pots (1.3 kg for size-6 pots), the proportion of nitrogen in the fertilizer (0.21 for ammonium sulphate), and the desired application rate. The final weights of ammonium sulphate were 0.186 g pot^−1^ to simulate 60 kg N ha^−1^ and 0.464 g for 150 kg N ha^−1^. A third group of control pots received no fertilizer. Seed was initially germinated under humid, dark conditions. After 3 days, healthy seedlings (seedling stage S3, where the prophyll had emerged from the coleoptile: Counce et al., 2000) of IR62 and IR22 were sown to the pots (one per pot) at staggered intervals such that host plants of three ages (15, 30 and 45 days after sowing [DAS]) were available at one time. These three ages were selected to correspond to pre-tillering plants (15 DAS: V3-V4 as classified by Counce et al., 2000), early tillering plants (30 DAS: >V4 and before the initiation of reproduction) and mid tillering plants (45 DAS: this corresponded with panicle initiation under greenhouse conditions, but was close to maximum tillering and before panicle initiation in all subsequent experiments, see also Counce et al., 2000). The pots were maintained in flooded steel trays to maintain soil saturation and to buffer against temperature extremes (Passioura, 2006, Crisol et al., 2013). Plants were monitored daily and pots were weeded as necessary. Plants were not treated with any pesticides. Once plants of the desired ages were available, planthopper nymph and adult performance was evaluated using a series of bioassays each replicated six times in a completely randomized design. The bioassays were conducted as follows:

Honeydew production was examined by infesting plants of each age and at each nitrogen level with two pre-starved (48 h) gravid females in specially prepared feeding chambers. The plastic chambers confined the adults to within 5 cm of the base of the plants and were placed over filter paper, neatly fitted around the plant shoot. The filter papers had been treated with bromocresol green to indicate the nature of the honeydew as coming from the phloem (basic) or xylem (acidic) (Horgan et al., 2016c). After feeding for 24 h, the area of excreted honeydew spots on the treated filter paper was measured using Image-J software version 1.48 (National Institute of Health, USA).

To examine nymph survival and development, 10 newly emerged nymphs were placed on rice plants (two plants per cage) of each age and under each nitrogen treatment. The plants were covered with acetate insect cages (40 × 10.5 cm; H × D) with mesh widows for ventilation. Nymphs were allowed to feed and develop for 10 days after which the number of survivors and their developmental stages were recorded. The insects were collected at the end of the experiment and the plants cut above the soil level. All materials were dried at 60 °C in a forced draught oven and weighed.

In a no-choice oviposition experiment, gravid females (one per plant) were placed on plants of each variety, at each plant age and under each nitrogen treatment. The plants were covered with acetate insect cages (as described above). Females were allowed to feed and lay eggs for 7 days after which they were removed and the plants dissected to count the eggs. The plants were cut above the soil and were dried in a forced draught oven at 60 °C before weighing.

#### 2.3.2 Screenhouse experiment

We evaluated the responses by IR62 and IR22 plants under three nitrogen levels when attacked by planthoppers at one of three plant ages. The screenhouse was covered by 1 mm mesh that restricted arthropod movement, but maintained normally cooler temperatures than in the greenhouse (26–35 °C; 12:12 h D:N). The IR62 and IR22 plants were grown in size-10 pots (22 cm × 24 cm: H × D) under zero-added nitrogen (0 kg N ha^−1^) or at 60 kg N ha^−1^ and 150 kg N ha^−1^. Nitrogen was added to the pots as ammonium sulphate. The amounts of fertilizer required were calculated for size-10 pots that contained 5.5 kg of soil. Equivalent weights were 0.786 g of ammonium sulphate to simulate 60 kg N ha^−1^, and 1.964 g for 150 kg N ha^−1^. Two thirds of the required fertilizer was applied prior to transplanting and the remainder applied at the tillering stage. Soil in the pots was maintained at saturation by daily watering, which buffered against temperature extremes. Plants were sown at one time and infested with planthoppers at 15, 30 or 45 DAS (corresponding to pre-tillering, early tillering and late tillering growth stages). To initiate infestations under similar densities of attack, gravid females were placed on the rice plants according to the biomass of the plants at each infestation date. Prior to infestation, two extra control plants of each variety were destructively sampled, dried in a forced draught oven and weighed to estimate shoot biomass. The gravid females were applied to the plants at a rate of one planthopper per gram of plant dry weight.

The planthoppers and plants were contained in mesh cages (160 × 22 cm: H × D). A set of corresponding, control (non-infested) plants was also planted and maintained under mesh cages until harvest (≥85% grain maturity). Plants were monitored daily and pots were watered and weeded as necessary. Plants were not treated with any pesticides. Severely planthopper-damaged plants were destructively sampled before final wilting. The planthoppers were first collected from the plants using a vacuum sampler (Hausherr’s Machine Works, USA) and were then dried, counted and weighed. Plants were then carefully pulled along with their roots and were washed, dried and weighed. Where plants had reached physiological maturity (i.e., ≥R7 – hard dough stage – according to classification in Counce et al., 2000), we noted grain production (weight), the number of filled grains, and the number of unfilled grains. Plants that did not wilt (normally the resistant lines and control, non-infested plants) were allowed to develop until harvest at which time they were destructively sampled and processed as indicated above. The bioassay was replicated six times in a randomized block design.

#### 2.3.3 Field experiment

A field study was conducted at IRRI Experimental Field Station (Los Baños, Laguna) during the 2011 wet season (WS) and the 2012 dry season (DS). The site had deep clay soils with about 4% organic matter. The experiment was conducted in a series of 18 rice plots of 33 × 12.5 m (L × W) that were each treated with one of three nitrogen regimes. These were zero-added nitrogen (0 kg N ha^−1^), 60 kg N ha^−1^ and 150 kg N ha^−1^. The nitrogen treatments were replicated in six fields, with each field containing three plots (i.e., 0, 60 and 150 kg N ha^−1^). Separate sub-irrigation channels were installed around each plot. These connected to the main field canals for irrigation and drainage but prevented leakage of nutrients among adjacent fields or among plots within each field.

At the time of the experiments, the fields were planted with IR66 (a variety reported to contain the *bph4* gene: Khush and Virk, 2005, Peñalver-Cruz et al., 2011). Seed was initially sown to dry seedbeds and transplanted as one plant per hill to the puddled field plots at 28 days after sowing (pre-tillering stage). Hills were spaced at a distance of 20 cm with 20 cm between planted rows (hill spacing = 20 cm × 20 cm). In the wet season (2011), four nitrogen (ammonium sulphate) top dressings were applied (basal, mid-tillering, panicle initiation and at one week before flowering). In the dry season (2012) three nitrogen (ammonium sulphate) top dressings were applied (basal, mid-tillering and before flowering). Solophos, muriate of potash and zinc were applied basally with the ammonium sulphate in each season. No pesticides were used in the fields at any time during the experiments.

During each season, four entomological field cages of 1 × 1 × 1.2 m (L × W × H) were installed in each of the plots. The cages were constructed of wooden frames and covered with 0.5 mm-bore insect netting. In June 2011 (wet season), IR62 rice plants were transplanted to two of the cages and IR22 to the remaining two cages as nine plants (hills) per cage arranged as three rows of three hills, each separated by 20 cm (hill spacing = 20 cm × 20 cm). The same set-up was repeated in 2012 (dry season) with transplanting conducted in January. Small plots such as these are considered adequate to assess yield and other plant characteristics in experiments such as ours where all plant genotypes used in the experiment have similar growth and development (i.e., short varieties with similar rates of maturation) (Jearakongman et al., 2003, Rebetzke et al., 2014).

Thirty days after transplanting (mid-tillering stage), plants inside two of the mesh cages (one with IR62 and one with IR22) in each plot were each infested with 25 second-instar planthopper nymphs (225 nymphs per cage). The remaining cages were left as non-infested controls. Plants were allowed to grow and develop until harvest, or until they showed symptoms of heavy planthopper damage (noted by severe yellowing of the leaves). Planthoppers inside the cages were collected when the plants were heavily damaged and before severe ‘hopperburn’ had killed any plants. Planthoppers were collected using a blow-vac suction sampler (Rice Vacuum Invertebrate Sampler – Poulan Pro-BVM200 LE, USA). At the same time that insects were collected, a single rice plant was randomly sampled from the same cage. In the remaining cages (control and surviving infested), planthoppers were collected at the time of harvest (≥85% grain maturity) and a single rice plant sampled at the same time.

After sampling, the numbers of tillers on each plant was recorded. The plants were then cleaned and divided into roots, shoots and panicles and the number of filled and unfilled grains recorded. All planthoppers and leafhoppers were separated from the complete arthropod samples. The planthoppers and leafhoppers were identified and dried separately (by species) in a forced draught oven at 60 °C for 3 days before being weighed. Plants, separated as roots, shoots and grain were also dried and weighed.

### 2.4 Data analyses

The effects of variety, plant age, nitrogen level and their interactions in the greenhouse experiment were examined using general linear models (GLM) with xylem-feeding, survival, planthopper biomass, biomass density, plant biomass change (per mg of planthopper), and eggs laid as dependent variables. Xylem-feeding was examined as a proportion of total honeydew production to standardize for planthopper biomass and different planthopper feeding rates (Horgan et al., 2016c). The covariate ‘total honeydew’ was initially included in the model, but was removed because it had no effect. Planthopper biomass per weight of plant was used as a metric for the severity of planthopper attack. Planthopper biomass has been shown to better represent planthopper populations on plants under varying nitrogen levels (Crisol et al., 2013). Plant biomass change per mg of planthopper was used as a metric of plant tolerance where biomass change = − (biomass of control plants – biomass of infested plants)/biomass of planthoppers (see Table 1). Biomass change as a function of planthopper biomass is influenced by intraspecific planthopper competition such that higher populations of planthoppers have lower individual effects on plant biomass (Horgan et al., 2016c). Because all nymphs had developed into adults on IR22, planthopper development in the greenhouse experiments was examined on IR62 only, using a two-way univariate GLM (model = nitrogen + age + interaction).

Plant growth parameters (tiller number, shoot biomass, root biomass, proportion of grain filled and dry grain weight) recorded from control plants at the time of harvest in the screen-house experiment were examined using GLM (model = variety + nitrogen + interaction). Parameters for the infested plants were similarly examined, but included the factor ‘time of infestation’. We estimated the impact of planthopper populations on shoot biomass, root biomass and grain yield in their respective plants as ‘biomass change’ (calculated as above). Biomass changes were analysed using two-way GLM.

Plant parameters (tiller number, shoot biomass, root biomass, proportion of grain filled, and dry grain weight) and herbivore parameters (biomass densities of brown planthoppers, whitebacked planthoppers and green leafhoppers) from the field experiments were examined using split–split plot GLMs with nitrogen as the main plot and variety and infested/control treatments as subplots. Root, shoot and grain biomass changes and changes in tiller number as a function of planthopper biomass were calculated as above (biomass change). Due to natural infestation of the control plants, planthopper biomass was calculated as the difference between the biomass of planthoppers in the infested cages and the biomass in corresponding control cages for each variety, nitrogen level and season. This assumes that brown planthoppers had the greatest overall effect on the rice plants and that whitebacked planthoppers and green leafhoppers had similar individual effects with brown planthoppers on the plants. We believe that this latter assumption would have little consequence to the overall results because of relatively low numbers of the latter two species in the infested cages. Biomass changes were not estimated for IR22 plants because plants often had severe yellowing or ‘hopperburn’ (plant desiccation and death due to planthopper feeding) soon after insect sampling and consequently high levels of intraspecific competition and declining planthopper fitness that prevented meaningful estimates of tolerance. Therefore, biomass changes were examined only for IR62 using univariate GLM with nitrogen as the independent variable and removing the effect of fields (blocks).

Residuals were plotted following all analyses. Where data were not normal and homogenous we applied appropriate transformations (indicated together with the results below). Where data could not be normalized we ranked cases. Analyses were conducted using SPSS v.22 (IBM SPSS, Armonk, NY, USA).

## 3 Results

### 3.1 Greenhouse experiments: ontogenetic changes in resistance and tolerance

Planthoppers produced more xylem-derived honeydew, had lower survival, slower development, lower biomass, and laid fewer eggs on IR62 than on IR22 (Table 2). Survival, egg-laying and biomass density declined on older plants, mainly due to increased resistance in IR62; this was further indicated by a significant [variety × plant age] interaction for xylem-derived honeydew, survival, biomass, and eggs laid (Table 2). Nitrogen increased planthopper survival, development (on IR62), planthopper biomass, and the number of eggs laid. There was a significant [variety × nitrogen] interaction for xylem-derived honeydew because of similar amounts produced by planthoppers feeding on both varieties under high nitrogen (150 kg N ha^−1^), but higher excretion of xylem-derived honeydew by planthoppers on IR62 under low nitrogen levels (0 and 60 kg N ha^−1^) – indicating an improvement in feeding efficiency (Table 2). There were significant [nitrogen × plant age] interactions for survival, development, and egg-laying, mainly because high nitrogen increased these fitness parameters on 45 DAS IR62 (thereby reducing resistance).

**Table 2:** Results from greenhouse bioassays on the effects of rice variety, nitrogen level and plant age on *Nilaparvata lugens* fitness parameters.

Variety	Nitrogen level (kg ha^−1^)	Plant age (DAS)	Xylem as a proportion of total honeydew	Nymph survival (%)	Nymph development (% adults)^a^	Nymph biomass (mg plant^−1^)	Number of eggs per plant
IR22							
	0	15	0.16 (0.07)	68.33 (12.24)	100.00 (0.00)	7.13 (1.77)	89.33 (20.02)
	0	30	0.01 (0.01)	85.00 (5.00)	100.00 (0.00)	8.54 (0.80)	140.67 (24.77)
	0	45	0.34 (0.21)	76.67 (7.15)	100.00 (0.00)	7.21 (1.02)	176.75 (14.10)
	60	15	0.17 (0.13)	65.00 (15.44)	100.00 (0.00)	6.12 (1.81)	152.67 (16.38)
	60	30	0.01 (0.01)	76.67 (16.06)	100.00 (0.00)	10.92 (2.61)	161.00 (18.63)
	60	45	0.06 (0.05)	80.00 (6.83)	100.00 (0.00)	10.09 (0.79)	144.20 (18.63)
	150	15	0.22 (0.10)	65.00 (9.57)	100.00 (0.00)	6.53 (1.52)	123.80 (21.28)
	150	30	0.04 (0.04)	91.67 (5.43)	100.00 (0.00)	11.03 (0.86)	179.00 (27.75)
	150	45	0.18 (0.18)	95.00 (3.42)	100.00 (0.00)	13.24 (0.55)	145.00 (26.85)

IR62							
	0	15	0.70 (0.18)	88.33 (11.95)	100.00 (0.00)	6.44 (0.86)	105.00 (17.38)
	0	30	0.80 (0.20)	83.33 (7.15)	89.68 (6.55)	4.79 (0.97)	86.00 (25.81)
	0	45	0.89 (0.11)	15.00 (9.57)	11.11 (11.11)	0.24 (0.18)	42.50 (9.92)
	60	15	0.75 (0.16)	78.33 (8.72)	95.83 (4.17)	5.90 (0.50)	154.50 (19.50)
	60	30	0.77 (0.15)	36.67 (11.16)	65.95 (17.21)	2.08 (0.71)	88.40 (20.51)
	60	45	0.24 (0.19)	26.67 (12.29)	21.88 (12.88)	0.67 (0.34)	87.75 (22.80)
	150	15	0.08 (0.08)	83.33 (5.58)	100.00 (0.00)	7.65 (0.87)	102.00 (30.00)
	150	30	0.51 (0.21)	86.67 (9.55)	97.22 (2.78)	4.20 (0.68)	130.50 (32.40)
	150	45	0.31 (0.13)	56.67 (15.20)	76.17 (12.96)	3.27 (1.63)	68.50 (9.25)
			Rank	arcsine	None	Rank	lg(sqrt)
F-variety (V)	1		59.749***	11.229***		82.524***	62.158***
F-nitrogen (N)^b^	2		2.468 ns	5.103**	38.240***	4.746*	6.592**
F-age (A)^b^	2		1.264 ns	5.611**	10.846***	1.396 ns	5.356**
F-V × N^b^	2		4.401*	1.214 ns		1.271 ns	1.269 ns
F-V × A	2		8.357***	16.167***		22.944***	19.082***
F-N × A^b^	4		1.621 ns	2.643*	4.556**	1.811 ns	2.987*
F-V × N × A^b^	4		1.569 ns	0.786 ns		0.651 ns	3.834**
Error	90						

a denominator df = 38.

b ns = P > 0.05.

* =P ≤ 0.05.

** =P ≤ 0.01.

*** =P ≤ 0.001.

**Fig. 1:**
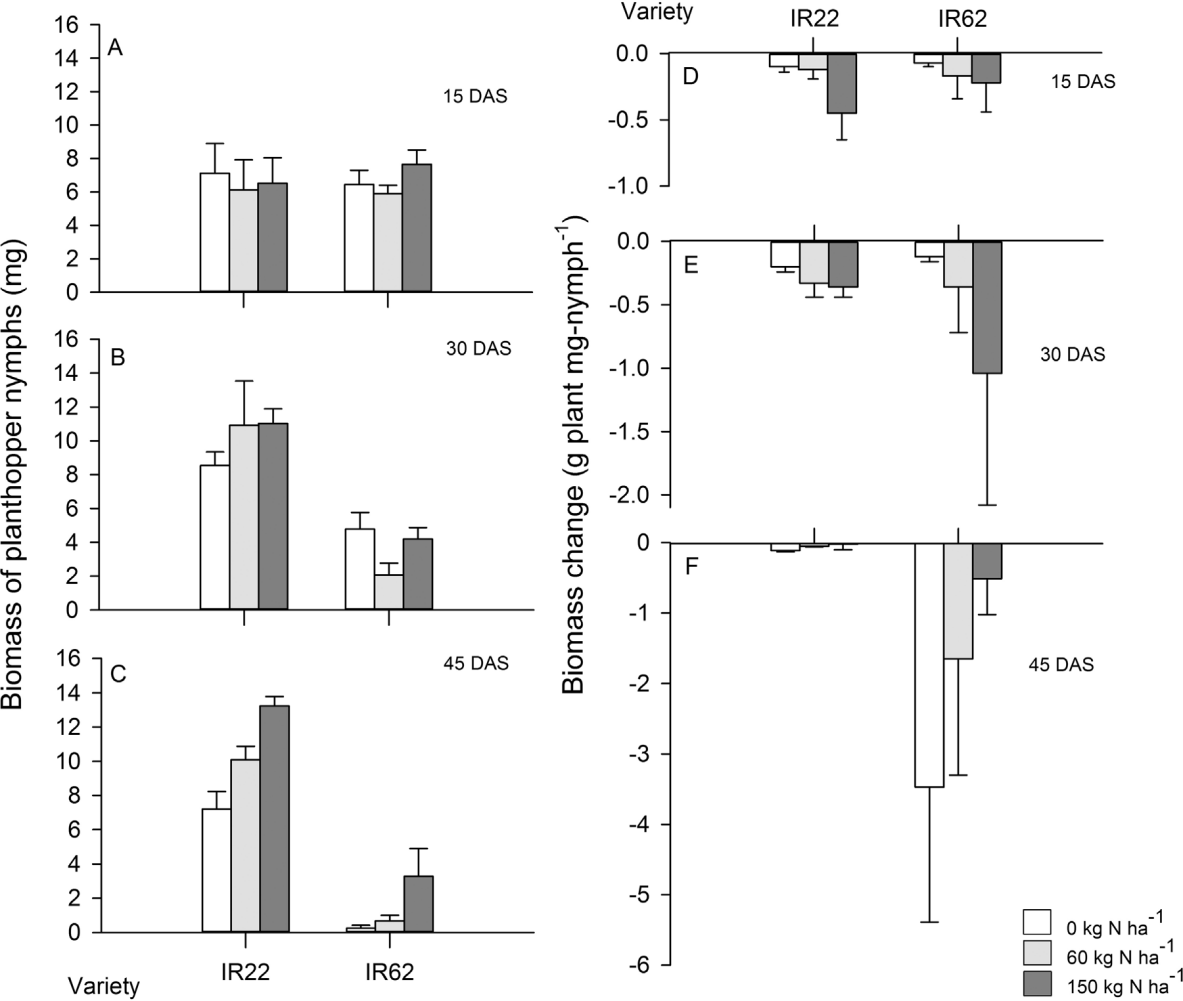
Biomass density of *Nilaparvata lugens* nymphs on susceptible (IR22) and resistant (IR62) rice plants. Rice plants were grown under three levels of nitrogen (0, 60 and 150 kg N ha^−1^) for 15, 30 or 45 days (corresponding to pre-tillering, early tillering and late tillering plant stages, respectively) before simultaneous infestation with planthoppers. Plants with 0 added nitrogen had low, residual nitrogen levels in the soil that were not determined during the bioassays. Standard errors are indicated (N = 6).

Planthopper biomass density (which indicates the net effects of nitrogen on planthopper-infestations under varying host plant conditions) was lower on IR62 (F_1,90_ = 64.166, P ≤ 0.001), declined under increasing plant age (F_2,90_ = 74.220, P ≤ 0.001) and under increasing nitrogen (F_2,90_ = 3.424, P = 0.037) (Fig. 1A–C). The effect of plant age was greater in IR62, resulting in a significant [variety × plant age] interaction (F_2,90_ = 5.952, P = 0.004). A significant [nitrogen × plant age] interaction for biomass density (F_4,90_ = 2.875, P = 0.027) was due to lower densities on 30 and 45 DAS plants under high nitrogen, but similar densities across nitrogen levels for 15 DAS plants (Fig. 1A–C). All other sources of variation were non-significant.

Planthoppers had a greater impact on the final biomass of IR62 plants than IR22 plants (Fig. 1D–F) (F_1,90_ = 2.662, P ≤ 0.001) suggesting that IR22 had higher tolerance to planthopper feeding in these short duration bioassays. Planthopper impact declined on IR22 plants as these aged, but increased on IR62 with age producing a significant [variety × plant age] interaction (F_2,90_ = 1.750, P ≤ 0.001). There was also a significant [nitrogen × plant age] interaction (F_2,90_ = 1.750, P ≤ 0.001) because high nitrogen increased tolerance in 45 DAS plants, but did not significantly affect tolerance in 15 and 30 DAS plants (Fig. 1D–F). All other sources of variation were non-significant.

### 3.2 Screenhouse experiment: biomass and yield loss in rice infested at different plant stages

Details of the growth parameters for control plants under varying nitrogen levels are presented in Supplementary Table S1. Details of planthopper biomass density and plant growth parameters in the infested pots are presented in Supplementary Table S2.

**Fig. 2:**
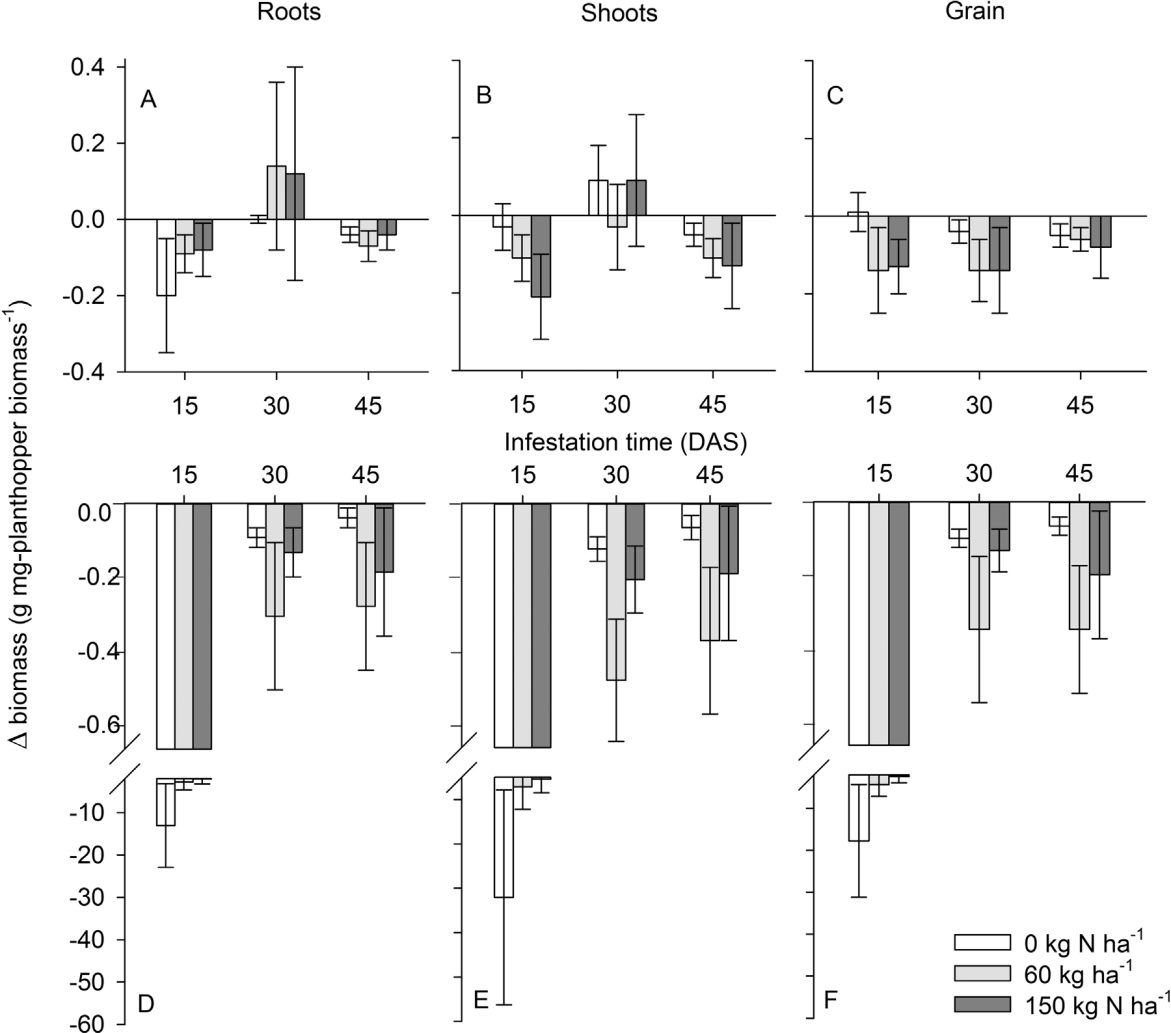
Changes (Δ) in root biomass (A,D), shoot biomass (B,C) and grain biomass (C,D) per mg of *Nilaparvata lugens* biomass on IR62 (A,B,C) and IR22 (D,E,F) under three nitrogen levels and infested with a constant density of planthoppers at 15, 30 and 45 DAS (corresponding to pre-tillering, early tillering and late tillering plant stages, respectively). Details of plant growth and yields in the experiment are available in Supplementary Tables S1 and S2. Standard errors are indicated (N = 6).

**Fig. 3:**
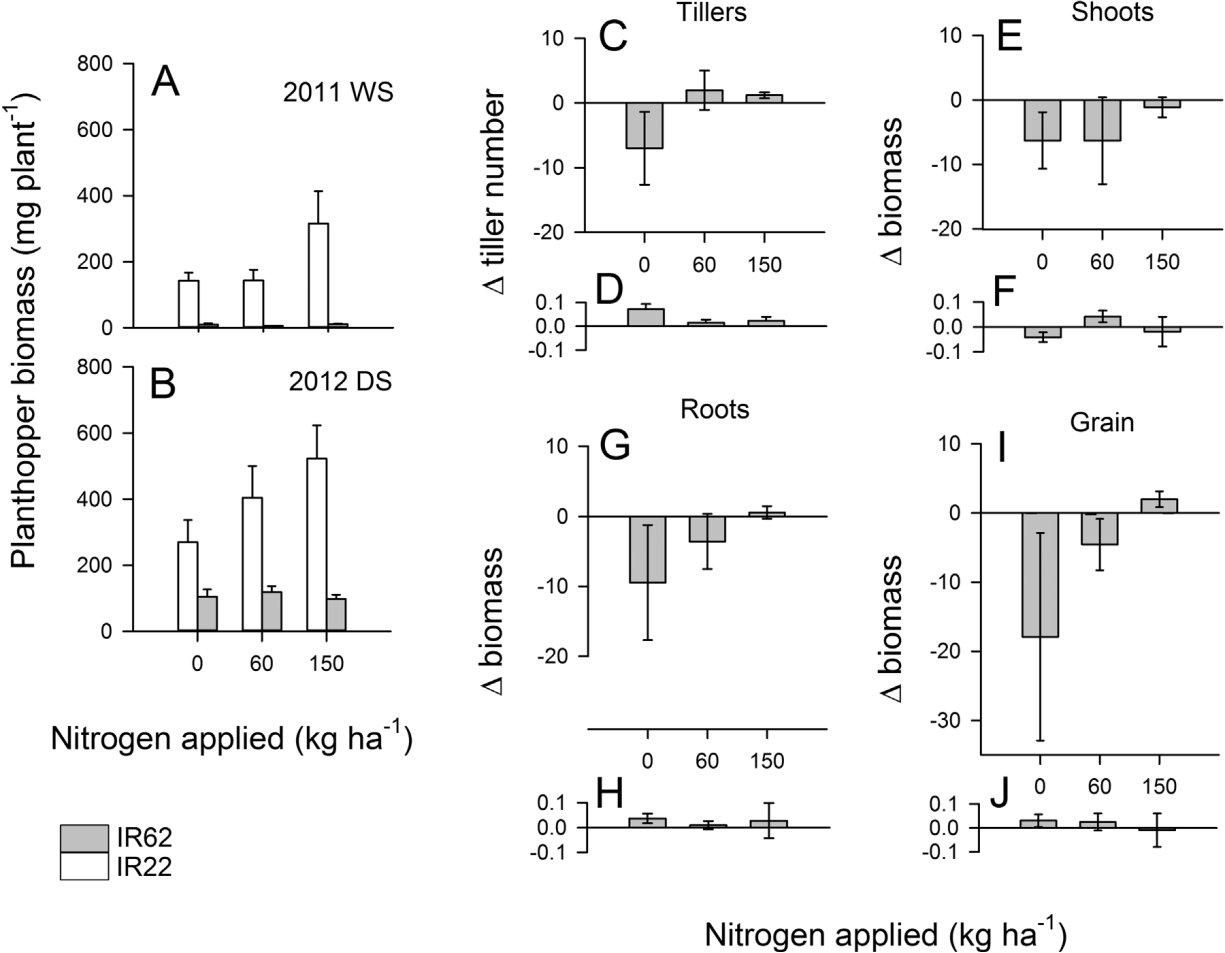
Total planthopper biomass in infested field cages with IR22 (open bars) and IR62 (shaded bars) rice plants during (A) the 2011 wet season and (B) the 2012 dry season. Changes (Δ) in plant tiller number and biomass (g-plant mg-planthopper^−1^) resulting from planthopper infestations were estimated for IR62 plants in 2011 (C,E,G,I) and 2012 (D,F,H,J). Changes in tiller number (C,D), shoot biomass (E,F), root biomass (G,H) and grain yield (I,J) under three levels of nitrogen fertilizer are presented. Standard errors are indicated (N = 6).

The negative impact of planthoppers on yield (grain: F_1,74_ = 17.812, P ≤ 0.001), shoot biomass (F_1,74_ = 29.474, P ≤ 0.001) and root biomass (F_1,74_ = 9.473, P = 0.003) was consistently higher on IR22 compared to IR62 (Fig. 2). Biomass losses to shoots (F_2,74_ = 7.403, P ≤ 0.001) and roots (F_2,74_ = 7.246, P ≤ 0.001) per mg of planthopper were consistently lower where plants had been infested at tillering stages (30 or 45 DAS: Fig. 3A,B,D,E). There were significant [variety × plant age] interactions for shoot (F_2,74_ = 5.506, P < 0.01) and root biomass loss (F_2,74_ = 3.404, P < 0.05) because of similar losses to plants of both varieties at older plant stages, but higher losses to IR22 plants infested at 15 DAS (pre-tillering) compared to IR62 plants infested at the same age (Fig. 3A,B,D,E). All other sources of variation were non-significant.

### 3.3 Field experiment: resistance and tolerance to planthoppers under varying nitrogen

Field cages were naturally infested with low densities of whitebacked planthoppers and green leafhoppers as well as wild brown planthoppers. Natural infestations were lower on IR62 than on IR22 and densities of each of the three species were correlated among treatments (variety and nitrogen level: Fig. S1). Further details of the planthopper and leafhopper densities are presented in Supplementary Fig. S1.

In the field experiments, IR62 had a higher number of tillers and a higher proportion of filled grain and attained greater grain yields than IR22 (Table 3, Table 4). IR62 also attained greater shoot and root biomass than IR22 during the 2011 wet season (Table 3). This higher performance of IR62 was mainly due to lower planthopper densities than on IR22 in the infested treatment. Infested plants had lower tiller numbers and lower shoot, root and grain biomass in 2011 (Table 3). In 2012, the infested plants had lower grain biomass and a lower proportion of filled grain (Table 4). Significant [variety × infestation] interactions in both years indicated that in most cases varietal performance was similar where plants had not been infested with planthoppers (tillers [2011,2012], root biomass [2011], filled grain [2011,2012], grain yield [2011,2012]) (Table 3, Table 4).

**Table 3:** Rice growth parameters at time of harvest (including plants that had died) from field exposures conducted during the 2011 wet season. Planthopper biomass density is indicated from field cages infested with laboratory reared *Nilaparvata lugens* (infested) and cages colonized by wild *N. lugens* (control).

Treatment (infestation and variety)	Nitrogen level (kg ha^−1^)^a^	Planthopper biomass density (mg g-plant^−1^)^b^	Number of tillers^b^	Shoot biomass (g dry weight)^b^	Root biomass (g dry weight)^b^	Percentage filled grain^b^	Biomass of filled grain (g dry weight)^b^
Control							
IR22	0	0.07 (0.02)	18.00 (1.86)	17.78 (2.68)	5.69 (1.24)	75.36 (15.14)	23.37 (5.43)
	60	0.13 (0.08)	24.33 (1.58)	21.83 (5.03)	6.30 (1.66)	80.61 (4.04)	22.53 (2.70)
	150	0.13 (0.09)	27.83 (3.20)	36.58 (9.28)	12.05 (3.72)	76.70 (3.70)	30.30 (5.64)
IR62	0	0.01 (0.01)	21.50 (2.31)	24.94 (2.91)	6.00 (0.83)	78.63 (2.42)	32.84 (5.07)
	60	0.04 (0.03)	22.50 (2.83)	28.09 (6.48)	5.92 (1.51)	81.64 (2.31)	31.35 (4.46)
	150	0.02 (0.01)	23.33 (4.02)	43.04 (9.13)	12.95 (2.94)	77.51 (8.34)	31.85 (6.46)

Infested							
IR22	0	6.86 (1.95)	7.00 (3.27)	12.00 (2.32)	2.72 (0.64)	52.66 (16.36)	3.67 (2.72)
	60	5.47 (1.29)	3.83 (3.83)	14.35 (4.27)	3.13 (0.80)	57.76 (0.00)	1.24 (1.24)
	150	7.34 (2.54)	5.67 (4.27)	22.40 (5.07)	5.83 (0.87)	37.32 (21.36)	2.10 (1.94)
IR62	0	0.27 (0.26)	15.50 (1.52)	23.01 (5.33)	4.96 (0.74)	84.59 (2.00)	22.81 (3.90)
	60	1.18 (1.14)	27.83 (4.56)	19.83 (3.22)	6.00 (1.10)	82.01 (3.44)	26.13 (7.04)
	150	0.06 (0.03)	35.00 (4.09)	38.82 (4.57)	9.03 (2.25)	82.27 (1.22)	50.44 (6.86)
F-nitrogen (N)^c^		0.006 ns	0.893 ns	2.938 ns	0.383 ns	0.245 ns	0.667 ns
(Linear contrast)^c^		0.031 ns	4.910**	16.160***	2.106*	1.030 ns	3.668*
F-control/infested (I)^c^		18.625***	7.277*	4.844*	6.754*	3.796 ns	8.103**
F-variety (V)^c^		14.653**	13.915**	5.314*	5.319*	8.021*	23.517***
F-N × V^c^		0.319 ns	0.550 ns	0.169 ns	0.454 ns	0.482 ns	0.692 ns
F-N × I^c^		0.021 ns	0.135 ns	0.296 ns	0.675 ns	0.303 ns	0.655 ns
F-V × I^c^		13.808**	16.716***	0.583 ns	5.181*	6.520*	9.828**
F-N × V × I^c^		0.313 ns	2.657 ns	0.103 ns	0.952 ns	0.488 ns	2.206 ns

Nominator df = 2 (nitrogen – main plot), 1 (infested/control), 1 (variety), 2 (N × V), 2 (N × I), 1 (V × I), 2 (N × V × I); denominator df = 71 (split–split plot design).

a Plants with 0 added nitrogen had low residual, background nitrogen levels in the soil. These were not determined during the experiment.

b Numbers in parentheses are standard errors (N = 6).

c ns = P > 0.05.

* =P ≤ 0.05.

** =P ≤ 0.01.

*** =P ≤ 0.001.

**Table 4:** Rice growth parameters at time of harvest (including plants that had died) from field exposures conducted during the 2012 dry season. Planthopper biomass density is indicated from field cages infested with laboratory reared *Nilaparvata lugens* (infested) and cages colonized by wild *N. lugens* (control).

Treatment (infestation and variety)	Nitrogen level (kg ha^−1^)^a^	Planthopper biomass density (mg g-plant^−1^)^b^	Number of tillers^b^	Shoot biomass (g dry weight)^b^	Root biomass (g dry weight)^b^	Percentage filled grain^b^	Biomass of filled grain (g dry weight)^b^
Control							
IR22	0	0.17 (0.05)	12.50 (0.89)	20.87 (3.80)	5.20 (1.42)	88.80 (2.03)	13.29 (2.33)
	60	0.23 (0.22)	19.67 (1.74)	25.65 (3.28)	11.36 (2.63)	82.28 (3.21)	23.42 (3.05)
	150	0.30 (0.26)	20.00 (1.46)	36.57 (5.08)	13.62 (3.22)	70.63 (8.37)	19.80 (3.54)
IR62	0	0.09 (0.09)	12.50 (1.26)	17.65 (1.96)	4.78 (0.96)	75.72 (4.07)	14.04 (1.66)
	60	0.20 (0.11)	19.50 (1.06)	26.46 (2.74)	8.70 (1.52)	78.79 (1.27)	19.65 (3.32)
	150	0.11 (0.09)	26.00 (0.06)	34.12 (4.60)	15.45 (3.31)	74.90 (5.45)	31.20 (5.36)

Infested							
IR22	0	174.12 (47.88)	8.17 (1.74)	16.11 (2.23)	2.53 (0.69)	25.23 (17.32)	1.47 (1.15)
	60	140.56 (33.60)	7.67 (3.52)	24.17 (4.68)	1.70 (0.79)	28.61 (14.35)	5.21 (2.50)
	150	139.59 (32.94)	9.50 (4.30)	31.26 (3.09)	6.15 (3.32)	27.41 (9.81)	2.92 (1.41)
IR62	0	52.17 (19.52)	16.67 (1.96)	14.94 (1.99)	7.04 (1.36)	68.86 (5.94)	15.64 (3.12)
	60	19.68 (12.57)	22.00 (1.41)	27.77 (2.76)	8.36 (1.19)	75.64 (4.71)	21.74 (1.68)
	150	31.04 (11.65)	27.33 (1.82)	29.00 (4.45)	14.20 (3.33)	72.12 (4.97)	30.74 (4.55)
F-nitrogen (N)^c^		0.126 ns	2.768 ns	4.037*	2.189 ns	0.199 ns	2.232 ns
(Linear contrast)^c^		0.694 ns	15.223***	12.281***	0.915 ns	15.223***	22.223***
F-control/infested (I)^c^		31.102***	3.579 ns	1.392 ns	3.496 ns	41.896***	10.273**
F-variety (V)^c^		12.441**	21.816***	0.093 ns	3.081 ns	21.364***	24.103***
F-N × V^c^		0.016 ns	1.819 ns	0.343 ns	0.325 ns	0.362 ns	3.487*
F-N × I^c^		0.383 ns	0.849 ns	0.357 ns	0.776 ns	0.616 ns	0.232 ns
F-V × I^c^		12.397**	12.242**	0.107 ns	3.995 ns	30.570***	13.539**
F-N × V × I^c^		0.017 ns	0.274 ns	0.023 ns	0.144 ns	0.257 ns	0.189 ns

Nominator df = 2 (nitrogen − main plot), 1 (infested/control), 1 (variety), 2 (N × V), 2 (N × I), 1 (V × I), 2 (N × V × I); denominator df = 71 (split–split plot design).

a Plants with 0 added nitrogen had low residual, background nitrogen levels in the soil. These were not determined during the experiment.

b Numbers in parentheses are standard errors (N = 6).

c ns = P > 0.05.

* =P ≤ 0.05.

** =P ≤ 0.01.

*** =P ≤ 0.001.

Although non-infested plants also had natural colonisation by planthoppers, the densities were similarly low on both IR62 and IR22 in the non-infested treatment (≤0.3 mg g-plant^−1^: Table 3, Table 4). Tiller number, shoot biomass and grain yield increased linearly with increasing nitrogen levels in both 2011 (Table 3) and 2012 (Table 4). Root biomass increased linearly with nitrogen during both seasons (significant effect in 2011: Table 3) and the percentage of grain filled tended to increase with nitrogen during the 2012 season (Table 4). During 2012, IR62 responded better to nitrogen as indicated by a significant [nitrogen × variety] interaction for grain yield in that season (Table 4).

Total planthopper biomass was higher on IR22 than IR62 in the artificially-infested field cages during both years (log + 1 transformed data: 2011 WS, F_1,23_ = 117.242, P = 0.008; 2012 DS, F_1,23_ = 18.454, P = 0.050) (Fig. 3A,B). Biomass density was not significantly affected by nitrogen levels (2011 WS, F_2,23_ = 1.127, P = 0.361, 2012 DS, F_2,23_ = 0.376, P = 0.696) and interactions were not significant (2011 WS, F_2,23_ = 0.739, P = 0.490; 2012 DS, F_2,23_ = 0.835, P = 0.448). Plants infested with planthoppers had lower tiller numbers, lower root and shoot biomass, and yielded less grain (Table 3, Table 4). High densities of planthoppers on IR22 in both years led to high tiller mortality (83%), reduced plant growth and low yields – yields of planthopper infested IR22 were generally lowest in the high nitrogen plots (Table 3, Table 4). In contrast, increasing nitrogen levels resulted in increasing tiller numbers (2011, 2012), greater root (2011) biomass and higher yields (2011, 2012) in planthopper-infested IR62 during both seasons (Table 3, Table 4).

During the 2011 wet season, planthopper populations on IR62 were low and the effects of planthoppers in reducing tiller number (ranked data, F_2,15_ = 3.401, P = 0.061, linear contrast P = 0.021: Fig. 3C), root biomass (ranked data, F_2,15_ = 2.370, P = 0.128, linear contrast P = 0.048: Fig. 3D) and grain weight (ranked data, F_2,15_ = 4.330, P = 0.033, linear contrast P = 0.010: Fig. 3F) declined significantly with increasing nitrogen levels. Reductions in shoot biomass due to planthoppers also tended to decline under high nitrogen, but the effect was not significant (ranked data, F_2,10_ = 0.099, P = 0.906, linear contrast P = 0.725: Fig. 3D). During 2012, relatively high planthopper densities on IR62 compared to 2011 (e.g., over 500 × in the high nitrogen treatment) reduced the impact of individual planthoppers (or unit weight of planthoppers) on final plant biomass and obscured treatment effects. Consequently, estimates of the effects of planthopper populations on IR62 tiller numbers, root and shoot biomass, and grain weight was low irrespective of nitrogen level (ranked data, 3.708 ≥ F_2,15_ ≥ 0.110, 0.062 ≥ P ≥ 0.896).

## 4 Discussion

This study describes an exception to the conventional understanding of brown planthopper-rice interactions (Sogawa, 1982, Horgan et al., 2016a, Hu et al., 2016). In our greenhouse experiment, adding nitrogen to rice at pre-tillering and early tillering stages resulted in lower tolerance to brown planthoppers in the susceptible (IR22) and resistant (IR62) varieties, corroborating results from a previous study with a range of susceptible rice varieties (Horgan et al., 2016a). The present study also indicated clear ontogenetic changes in tolerance, as well as ontogenetic changes in tolerance responses to nitrogen. Tolerance increased in older IR22 plants, but decreased in older IR62 plants; however, high nitrogen applied to these older IR62 plants increased their tolerance to planthopper (nymph) feeding. A similar trend was noted during our 2011 field experiment: planthopper effects on tiller number, root biomass and grain yield all declined significantly under high nitrogen, indicating that the plants gained tolerance to the planthoppers under increasing nitrogen.

When tested in the greenhouse, nitrogen generally improved the fitness of planthoppers on IR62. However, biomass density either declined, or remained constant under increasing nitrogen. Similar trends were noted in the screenhouse experiment and in the 2011 and 2012 field experiments. These results suggested that the phloem of IR62 plants can support only low numbers of planthoppers, resulting in strong intraspecific competition between planthoppers even at low and generally non-damaging densities. These results support those from a related study where the mortality of planthoppers on IR62 increased rapidly under increasing conspecific density (Horgan et al., 2016c). Furthermore, the results of this study suggest that planthopper resistance in rice may have added benefits for farmers through increased tolerance under high nitrogen. In contrast, in susceptible varieties, high nitrogen is often associated with increased mortality of planthopper infested plants (Horgan et al., 2016a).

### 4.1 Caveats and caution in interpreting results

Research into the ontogenetics of plant resistance and tolerance to herbivores requires the careful manipulation of herbivore pressure during discrete plant stages. Such controlled experiments are perhaps best conducted in greenhouses or climate chambers using potted plants. Our results from the greenhouse bioassays show clear ontogenetic shifts in resistance on IR62 (increasing on older plants) and in tolerance on both IR22 and IR62 (increasing and decreasing on older plants, respectively). Furthermore, nitrogen clearly reduced resistance in IR62 and reduced tolerance in young IR62 and IR22 plants (15–30 DAS) but increased tolerance in older IR62 plants (45 DAS). Infestations of rice plants by planthoppers, and other herbivores, are not restricted to discrete life stages; indeed planthoppers can have up to three generations on a single rice plant (Bottrell and Schoenly, 2012, Horgan et al., 2016a). Our screenhouse experiment indicated that infestation of pre-tillering rice plants leads to greater loses in biomass and yield (as in IR22). Resistance in IR62, which increases at older plant stages reduced the impact of the planthoppers to similar levels regardless of the stage at which the plants were initially infested.

Experiments using potted plants have been noted elsewhere as limited in their usefulness for making predictions about field situations, particularly where fertilizers are included as a treatment (Passioura, 2006, Poorter et al., 2012, Crisol et al., 2013). We took precautions during our greenhouse and screenhouse experiments by using pots that were large enough to produce rice plants without appreciably reducing their above-ground biomass when compared to field-grown pure-line varieties (Crisol et al., 2013). However, even in large pots, yield is normally reduced compared to field-grown rice plants and insect cages will further reduce final yields (Crisol et al., 2013). Experiments conducted in field plots are more representative of farmers’ fields, particularly where the plots are used to compare yields among morphologically similar rice varieties as in the present study (Jearakongman et al., 2003; Rebetzke et al., 2014). Our 2011 WS and 2012 DS field experiments successfully demonstrated that the resistant variety IR62 had higher root, shoot and grain yields in high nitrogen plots despite planthopper infestations. The reductions in resistance that we noted in the greenhouse under high nitrogen, did not translate into appreciably higher planthopper densities in high nitrogen field plots. Furthermore, a positive effect of high nitrogen on tolerance in IR62, which had been previously noted in the greenhouse study, was apparent from our 2011 WS field experiment.

Higher tolerance to planthopper feeding at reproductive stages − including during grain-filling, will be particularly beneficial to rice farmers. During the last two decades, tolerance as a concept in herbivory has gained increased attention. However, several confounding concepts of tolerance exist, including tolerance as a component of resistance (i.e., Ren et al., 2016), and tolerance as a feature of plant growth (i.e., Qiu et al., 2011). Because tolerance is a relative measure, it can only be assessed by measuring the plant’s fitness (biomass, yield, etc.) under more than one condition (i.e., herbivore damage or herbivore pressure) (Simms, 2000, Wise and Carr, 2008). Therefore, herbivory tolerance is often calculated as the slope of the regression of plant fitness against herbivore damage (Wise and Carr, 2008). Previous studies of planthopper tolerance in rice have used a variety of indices that relate normal growth and development of rice plants (under control conditions) to growth and development in infested plants (Ho et al., 1982, Panda and Heinrichs, 1984, Panda et al., 1984, Chen et al., 2003, Qiu et al., 2011). Such simplified metrics are useful for field experiments where treatment replications are often limited by high costs. Because planthopper feeding is not consistent during experiments with resistant and susceptible rice plants, we approximated tolerance by standardizing herbivore pressure. Whereas this reduces biases in tolerance estimates from contrasting plant types (susceptible/resistant or nitrogen gradients), it does not reduce the confounding effects of intraspecific competition under high population densities. As indicated by Horgan et al. (2016c), intraspecific competition can occur at relatively low densities among planthoppers feeding on resistant varieties. This suggests that tolerance estimates improve under lower planthopper densities.

### 4.2 Resistance in IR62

Stevenson et al. (1996) suggested that high concentrations of schaftoside, isoschaftoside, and total apigenin-C-glycosides (all are C-glycosidic flavonoids) in rice varieties (Rathu Heenati, BG300, and BG379/2) purported to contain the *Bph3* locus had an anti-feeding effect on planthoppers. In feeding tests, these authors found mortality of the planthoppers to increase as schaftoside concentrations increased from 250 to 500 μg mL^−1^. More recently, Ren et al. (2016) have indicated that the *Bph32* gene (from PTB33) encodes a protein similar to lectins that act on insect glycoproteins or tissues and inhibit insect feeding. The gene was highly expressed in the leaf sheaths of rice at 2 and 24 h after planthopper infestation, suggesting an induced response pathway (Ren et al., 2016). In the present study, planthoppers on IR62 excreted relatively large amounts of honeydew derived from nutrient poor xylem, they also had lower survival, attained a lower body weight and laid fewer eggs than on IR22. Xylem feeding may be a mechanism by which planthoppers remain hydrated where they fail to acquire enough fluids from the phloem (Pompon et al., 2011). We noted resistance in IR62 to increase as the rice plants aged. In particular, nymph development was significantly slower on 45 DAS (mid- to late tillering stages) IR62 plants. The durability of resistance in IR62 (now over 30 years since it was released in the Philippines: Peñalver-Cruz et al., 2011) contrasts with the rapid adaptation by planthoppers to other deployed genes (i.e., *Bph1* and *bph2/BPH26*: Myint et al., 2009, Horgan et al., 2015). It also appears that farmer adoption of IR62 and other varieties with resistance derived from PTB33 or Rathu Heenati may be low enough to avoid the selection of virulent planthoppers (Khush and Virk, 2005, Peñalver-Cruz et al., 2011). Our results suggest that, even if adoption has been low, the specific mechanisms of resistance associated with IR62, the stability of these mechanisms under high nitrogen conditions (and presumably where the nutritional quality of the phloem had improved) and the interactions between resistance and plant tolerance will likely promote high durability of this particular resistance source. Nevertheless, the deployment of varieties with PTB33 or Rathu Heenati as resistance donors should be limited temporally and spatially to avoid largescale planthopper adaptation to genes associated with the *Bph3* locus.

### 4.3 Ontogenetic changes in resistance and tolerance

Ontogenetic changes in resistance to insect herbivores are common among plants and have been well documented for higher plants in particular (Boege and Marquis, 2005, Boege et al., 2007). Young leaves have comparatively high concentrations of nitrogen, a higher water content, and have lower carbon:nitrogen ratios compared to older leaves, making young leaves more attractive to many herbivores (Coley, 1980, Aide, 1993). Furthermore, in grasses such as rice, older plants will often accumulate silicon, which protects them from herbivores and diseases (Reynolds et al., 2009). Young, pre-tillering rice plants tend to be silicon deficient (Ma et al., 1989); therefore, pre-tillering rice should be expected to make a higher investment in anti-herbivore resistance particularly in varieties with major resistance genes. However, several studies suggest that this is generally not the case, but that older rice plants often become increasingly resistant to herbivores, particularly planthoppers and leafhoppers (Rapusas and Heinrichs, 1982, Baqui and Kershaw, 1993, Horgan et al., 2016b, Srinivasan et al., 2015, Srinivasan et al., 2016). The results from the present study confirm that pre-tillering plants are more susceptible than tillering rice plants, even in IR62. Increasing resistance to planthoppers in older rice may be related to thickening of the leaf sheath and stems, representing a physical barrier to planthopper feeding and oviposition. For example, oviposition by brown and whitebacked planthoppers often decreases as rice plants age, even on susceptible varieties (Horgan et al., 2016c). It is likely that such physical barriers increase the resistance of IR62 on older plants by working in synergy with phloem-based resistance factors.

The question remains as to why young rice plants should be less resistant to herbivores. One possibility is that in annual grasses such as rice, seedlings and actively growing tillers encounter herbivores, particularly specialists such as monophagous planthoppers, at relatively less damaging stages (i.e., early instars and early generations with consequently lower population densities). As these insects grow and develop, their damage potential increases, thereby representing a greater threat to older plant stages. Furthermore, early-tillering rice plants can often compensate well for herbivore damage by rerouting nutrients among tillers or by producing new tillers (Mae and Ohira, 1981, Rubia-Sanchez et al., 1999, Lv et al., 2008).

We expected that older IR22 and IR62 plants (at low nitrogen levels) would tolerate planthoppers by continuing to route nutrients to reproductive organs such as panicles and grain (Horgan et al., 2016a). In our screenhouse experiment, IR22 plants infested at older stages (when plants were tillering) had considerably higher tolerance than plants infested at 15 DAS (pre-tillering) although planthopper biomass had also built-up more on the plants infested at pre-tillering. But older IR62 plants were not appreciably more tolerant than younger plants, although the roots and shoots of IR62 plants infested at early tillering (30 DAS) tended to overcompensate for planthopper damage. The greater increase in tolerance in tillering IR22 (compared to pre-tillering IR22), but not in older IR62, was also apparent in the results of our greenhouse experiment where we noted increases in plant biomass loss per mg of planthopper on tillering IR62 plants despite decreasing planthopper biomass density. Decreasing tolerance in older IR62 plants is difficult to explain, but might be due to ecological trade-offs between resistance and either maintenance or growth (Leimu and Koricheva, 2006, Núñez-Farfán et al., 2007). Previous studies with other rice herbivores have indicated lower tolerance and greater effects on yield, particularly when older rice plants are attacked by stemborers, compared to attacks on earlier plant stages (Rubia et al., 1989). This appears to relate to a decreasing ability for rice plants to partition resources away from damaged tillers and toward new tillers during plant reproductive stages such that herbivore feeding competes directly with grain filling (Viajante and Heinrichs, 1987, Rubia et al., 1989, Rubia-Sanchez et al., 1999).

### 4.4 Stability of resistance and tolerance under high nitrogen

In our experiments, planthopper fitness often increased on high nitrogen plants, particularly during late tillering stages (45 DAS). Recent studies have indicated that anti-herbivore resistance associated with a range of different resistance genes is compromised by high nitrogen conditions (Salim and Saxena, 1991, Vu et al., 2014, Horgan et al., 2016b, Srinivasan et al., 2016). In the present study, planthopper nymphs developed faster and had greater survival on IR62 plants grown under high nitrogen. However, nymph biomass-density declined significantly on IR62 under increasing levels of nitrogen in the greenhouse bioassays. Increases in certain fitness parameters such as survival and development time during short-duration experiments might be trivial compared to changes in planthopper biomass (particularly among females) and egg-laying during population development. This was borne out in the screenhouse and field experiments, where planthopper biomass/biomass-density on IR62 did not respond to increasing nitrogen levels. In contrast to resistance, herbivore tolerance in IR62 clearly increased under higher nitrogen regimes during the 2011 field experiment and there was a tendency for overcompensation of root and grain biomass linked to high tillering as a response to planthoppers during 2012 (these results were supported by results from the greenhouse experiments). This apparent capacity of IR62 to better tolerate brown planthopper damage than IR22, and the possibility that tolerance to planthopper damage increases in IR62 under high nitrogen conditions contrasts with previous studies that indicate little or no tolerance to brown planthopper damage among field-grown rice plants, and increasingly severe damage due to brown planthoppers under high nitrogen (Cuong et al., 1997, Jiang and Cheng, 2003, Crisol et al., 2013, Horgan et al., 2016a). We believe that this difference may be attributed to the particular resistance genes or particular resistance mechanisms operating in IR62, since previous studies have only examined tolerance in susceptible varieties (Rubia-Sanchez et al., 1999, Srinivasan et al., 2015, Horgan et al., 2016a).

### 4.5 Implications for crop management

Our results indicate that farmers using high fertilizer applications on IR62 will not appreciably accelerate adaptation by planthoppers to the variety’s specific resistance genes. However, because IR62 is naturally infested by planthoppers in the field (as noted in our field experiments), there is potential for planthoppers to adapt to the variety (see Ferrater et al., 2015, for rates of planthopper adaptation on IR62). Careful management of planthopper populations by conserving natural enemies (Kenmore et al., 1984; Settle et al., 1996) and by avoiding resurgence pesticides (Gallagher et al., 1994, Heinrichs, 1994a, Heinrichs, 1994b) is still required to preserve the effectiveness and to prolong the durability of resistance in IR62 and related varieties. Finally, it has become increasingly clear that resistance genes do not work in isolation, but influence plants through networks of interacting genes (Fujita et al., 2013). Horgan et al. (2015) have indicated that some (but not all) rice varieties thought to possess the *Bph3* locus or the closely related *bph4* locus are no longer effective against the brown planthopper even in the Philippines −highlighting the role of associated rice genes and variety traits in enhancing resistance (see also Peñalver-Cruz et al., 2011). Furthermore, Jairin et al. (2007) have indicated that planthoppers in Thailand can cause serious damage to the rice variety Rathu Heenati with the *Bph3* locus, by attacking the developing rice panicles but avoiding shoots. These observations also indicate that host plant resistance in rice (and other crops) can only be considered as a component of integrated pest management and that a greater emphasis on ecological studies will be necessary to successfully deploy resistant rice varieties in modern rice production systems. Understanding not only resistance, but also tolerance to herbivores under varying crop management practices will help design improved crop production systems that support the natural regulation of crop pests.
