# Supplementary material for: Resistance and tolerance to the brown planthopper, Nilaparvata lugens (Stål), in rice infested at different growth stages across a gradient of nitrogen applications
Source: Field Crops Res. 2018 Mar;217:53–65. doi: 10.1016/j.fcr.2017.12.008 (PMC5777095; doi:10.1016/j.fcr.2017.12.008)
Supplement: Supplementary file 1 [file mmc1.docx]

**Supplementary information 1 – Development of control plants in screenhouse experiment**

Potted IR22 and IR62 plants had similar shoot and root biomass and produced a similar amount of filled grain by the time of harvest (Table S1). However, IR62 plants produced more tillers and had a higher proportion of unfilled grain (Table S1). Nitrogen affected tiller number, shoot biomass, root biomass and grain weight in both varieties, but had no effect on the proportion of seed with unfilled grain. However, there was a significant variety × nitrogen interaction, because at high nitrogen levels the proportion of filled grain in IR62 increased (Table S2).

Table S1 Growth parameters of IR22 and IR62 at time of harvest when produced in screenhouse pots (size 10) with equivalents of 0, 60 and 150 Kg/ha of added nitrogen.

| Variety | Nitrogen level (kg/ha)^1^ | Number of tillers^2,3^ | Shoot biomass (g dry weight)^2,3^ | Root biomass (g dry weight)^2,3^ | Proportion of grain filled^2^ | Biomass of filled grain (g dry weight)^2,3^ |
| --- | --- | --- | --- | --- | --- | --- |
| IR22 | 0 | 7.00 (0.58)A | 18.60 (0.46)A | 6.93 (1.61)A | 0.74 (0.04) | 10.13 (0.85)A |
|  | 60 | 8.00 (0.37)A | 22.57 (0.50)B | 7.68 (1.00)AB | 0.84 (0.01) | 12.81 (0.96)B |
|  | 150 | 10.67 (0.61)B | 32.28 (1.27)C | 12.07 (2.15)B | 0.79 (0.03) | 14.28 (0.88)C |
| IR62 | 0 | 7.67 (0.33) | 18.10 (0.92) | 6.42 (1.15) | 0.65 (0.03) | 9.86 (1.03) |
|  | 60 | 9.33 (0.49) | 21.70 (0.43) | 7.44 (1.40) | 0.60 (0.04) | 10.73 (0.86) |
|  | 150 | 12.83 (1.01) | 29.64 (1.90) | 10.66 (2.58) | 0.74 (0.04) | 15.73 (2.58) |
| F-variety (V) |  | 7.793** | 2.401ns | 0.259ns | 20.788*** | 0.073ns |
| F-nitrogen (N) |  | 27.643*** | 74.739*** | 4.115* | 2.012ns | 7.119** |
| F-V×N |  | 0.761ns | 0585ns | 0.062ns | 3.570* | 0.856ns |

1: Plants with 0 added nitrogen had low residual, background nitrogen levels in the soil. These were not determined during the experiment

2: Numbers in parentheses are standard errors (N = 6)

3: Letters indicate homogenous nitrogen groups (letters represent corresponding nitrogen levels throughout each indicated column)

4: ns = P > 0.05; * = P ≤ 0.05; ** = P ≤ 0.01, *** = P ≤ 0.001, nominator df = 1 (variety), 2 (nitrogen), 2 (V×N); denominator df = 30.

**Supplementary information 2 – Development of infested plants in screenhouse experiment**

Planthoppers attained a greater biomass density on the IR22 plants (Table S2). At the time of harvest, resistant plants infested with planthoppers had more live tillers, attained a greater shoot and root biomass, had a greater proportion of grain filled and had higher yields than planthopper-infested IR22 plants (Table S2). High nitrogen increased tillering and shoot biomass and resulted in higher grain yields (Table S2). Tiller number, shoot biomass and root biomass were significantly lower in plants that were infested as seedlings at 15 DAS than in plants infested at 30 or 45 DAS (PLANT STAGES); these effects were most apparent in infested IR22 plants under low nitrogen, resulting in significant variety × age (tillers, shoot biomass, root biomass) and variety × nitrogen (tillers) interactions.

Table S2 Rice growth parameters at time of harvest (including plants that had died). Rice plants were grown under three levels of nitrogen (0, 60 and 150 kg ha^-1^) and were infested with *Nilaparvata lugens* at 15, 30 or 45 days after sowing (DAS) (corresponding to pre-tillering, early tillering and maximum tillering rice stages, respectively). Data for control (non-infested plants) are presented in Supplementary Table S1

| Variety | Nitrogen level (kg ha^-1^)^1^ | Plant age (DAS) | Number of tillers/plant^2,3^ | Shoot biomass (g dry weight)^2,3^ | Root biomass (g dry weight)^2,3^ | Proportion of grains filled^2^ | Weight of filled grain (g dry weight)^2,3^ | Planthopper biomass density (mg/g plant)^2^ |
| --- | --- | --- | --- | --- | --- | --- | --- | --- |
| IR22 | 0 | 15 | 2.50 (0.43)Aa | 1.71 (0.42)Aa | 0.09 (0.09)a | 0.00 (0.00) | 0.00 (0.00)A | 22.71 (14.12) |
|  |  | 30 | 7.00 (0.63)b | 6.95 (0.87)b | 2.42 (0.64)b | 0.00 (0.00) | 0.00 (0.00) | 15.80 (3.64) |
|  |  | 45 | 7.67 (0.56)b | 12.16 (2.18)b | 4.70 (1.19)b | 0.28 (0.28) | 0.55 (0.55) | 15.93 (4.92) |
|  | 60 | 15 | 3.50 (0.34)B | 4.62 (1.05)B | 0.61 (0.27) | 0.00 (0.00) | 0.00 (0.00)AB | 26.02 (15.74) |
|  |  | 30 | 8.00 (1.30) | 8.90 (0.91) | 3.43 (0.84) | 0.00 (0.00) | 0.00 (0.00) | 17.48 (6.40) |
|  |  | 45 | 9.17 (0.91) | 13.91 (1.35) | 4.31 (1.44) | 0.00 (0.00) | 0.00 (0.00) | 10.15 (5.61) |
|  | 150 | 15 | 4.67 (1.69)C | 7.86 (2.89)C | 1.79 (1.07) | 0.64 (0.64) | 2.75 (2.75)B | 24.32 (12.78) |
|  |  | 30 | 5.83 (0.48) | 11.39 (1.40) | 3.22 (0.86) | 0.00 (0.00) | 0.00 (0.00) | 15.16 (4.95) |
|  |  | 45 | 11.00 (0.68) | 26.41 (6.22) | 6.46 (2.35) | 0.00 (0.00) | 0.00 (0.00) | 19.02 (4.89) |
| IR62 | 0 | 15 | 8.17 (0.48) | 17.07 (0.82) | 4.87 (0.80) | 0.65 (0.04) | 9.94 (0.80) | 1.69 (1.35) |
|  |  | 30 | 7.50 (0.81) | 17.88 (1.81) | 6.10 (0.75) | 0.54 (0.13) | 8.72 (2.16) | 2.60 (1.06) |
|  |  | 45 | 7.67 (0.49) | 14.31 (0.65) | 3.33 (0.43) | 0.59 (0.04) | 6.43 (0.61) | 6.79 (2.68) |
|  | 60 | 15 | 11.17 (2.17) | 18.64 (2.51) | 3.84 (0.96) | 0.34 (0.06) | 6.03 (1.86) | 5.97 (1.58) |
|  |  | 30 | 11.33 (0.95) | 22.01 (1.69) | 5.94 (1.55) | 0.53 (0.03) | 9.28 (0.74) | 1.44 (0.63) |
|  |  | 45 | 9.50 (0.56) | 16.38 (1.04) | 4.29 (0.63) | 0.62 (0.09) | 6.72 (1.02) | 3.00 (1.49) |
|  | 150 | 15 | 12.33 (0.92) | 19.52 (2.77) | 4.34 (1.46) | 0.50 (9.65) | 9.65 (2.22) | 4.22 (1.65) |
|  |  | 30 | 15.17 (0.87) | 27.56 (1.51) | 6.42 (1.82) | 0.55 (0.08) | 12.81 (1.93) | 0.93 (0.58) |
|  |  | 45 | 15.50 (1.54) | 22.63 (2.27) | 6.35 (1.10) | 0.55 (0.08) | 12.83 (2.17) | 12.52 (4.78) |
| Transformation | |  | None | None | None | Rank | Rank | None |
| F-variety (V)^4^ | |  | 84.789*** | 144.282*** | 15.162*** | 420.033*** | 415.223*** | 16.609*** |
| F-nitrogen (N)^4^ | |  | 24.351*** | 21.934*** | 1.976ns | 2.039ns | 4.637* | 0.132ns |
| F-age (A)^4^ | |  | 14.595*** | 14.820*** | 6.923** | 0.331ns | 0.742ns | 0.240ns |
| V×N^4^ |  |  | 10.284*** | 0.038ns | 0.063ns | 0.991ns | 1.433ns | 0.780ns |
| V×A^4^ |  |  | 11.052*** | 21.040*** | 5.811** | 0.663ns | 1.736ns | 0.556ns |
| N×A |  |  | 1.640ns | 1.015n | 0.491ns | 1.133ns | 0.390ns | 1.470ns |
| V×N×A |  |  | 1.944ns | 0.867ns | 0.333ns | 1.358ns | 2.001ns | 0.584ns |

1: Plants with 0 added nitrogen had low residual, background nitrogen levels in the soil. These were not determined during the experiment.

2: Numbers in parentheses are standard errors (n = 6)

3: Upper case letters indicate homogenous nitrogen groups, lower case letters indicate homogenous plant age groups (letters represent corresponding nitrogen and age levels throughout each indicated column)

4: ns = P > 0.05; * = P ≤ 0.05; ** = P ≤ 0.01, *** = P ≤ 0.001, nominator df = 1 (variety), 2 (nitrogen), 2 (age), 2 (V×N), 2 (V×A), 4 (N×A), 4 (V×N×A); denominator df = 89.

**Supplementary information 3 – Details of planthopper communities in field cages**

Plants under both the infested and non-infested treatments were colonized by wild planthoppers and leafhoppers in the field experiment. These consisted mainly of white-backed planthoppers and the green leafhopper. Densities of white-backed planthoppers were lower on IR62 compared to IR22 during 2011 (F_1,55_ = 10.275, P = 0.006; all other factors were non-significant) and densities of green leafhoppers were lower on IR62 than IR22 during both seasons (2011: F_1,55_ = 6.113, P = 0.26; 2012: F_1,55_ = 7.045, P = 0.018; all other factors were non-significant). Higher densities on IR22 were partly due to higher susceptibility of the variety to both herbivore species, but were also apparently due to facilitation by brown planthoppers as indicated by a significant positive correlations between the three main hopper species in the field cages (Figure S1).

Supplementary Figure S1 Correlations between *Nilaparvata lugens* densities on IR62 (open symbols) and IR22 (solid symbols) with *Sogatella furcifera* (A,D), and *Nephotettix virescens* (B,E) and between *S. furcifera* and *N. virescens* (C,F). Data were collected from field cages across all treatments during the 2011 WS (A,B,C) and 2012 DS (D,E,F). Pearson (indicated by ‘a’) and Spearman correlation coefficients are indicated. * = P ≤ 0.05; ** = P ≤ 0.01; *** = P ≤ 0.001 (N = 6).
